# Correlation of NT-proBNP With Duration of Hospital Stay in Patients With Heart Failure: A Retrospective Review

**DOI:** 10.7759/cureus.97784

**Published:** 2025-11-25

**Authors:** Harshitha Harshitha, Krishna Prasad Lakshminarayana Hoskere, Meghana Sreenivas, Prashanth YM

**Affiliations:** 1 General Medicine, Father Muller Medical College, Mangalore, IND; 2 Acute Medicine, Dorset County Hospital Foundation Trust, Dorchester, GBR; 3 General Medicine, BGS Medical College and Hospital, Bengaluru, IND; 4 General Medicine, Father Muller Medical College Hospital, Mangalore, IND

**Keywords:** biomarker, heart failure, hospital stay, mortality, nt-probnp, prognosis, retrospective study

## Abstract

Background: Heart failure (HF) is a leading cause of hospitalization and mortality worldwide, and the burden of the disease is increasing on a daily basis. N-terminal pro-B-type natriuretic peptide (NT-proBNP) is an established biomarker for diagnosis and prognosis in HF, but its relationship with length of hospital stay remains underexplored. Understanding this association may help in early risk stratification and optimize healthcare resource utilization.

Objective: To evaluate the correlation between NT-proBNP levels and length of hospital stay in patients admitted with HF and to identify clinical predictors of in-hospital mortality.

Methods: This retrospective observational study included 45 patients admitted with HF to Father Muller Medical College Hospital, Mangalore, between October 2024 and May 2025. Demographic, clinical, echocardiographic, and laboratory data, including NT-proBNP levels, were collected from hospital records. Statistical analyses were performed using Jamovi 2.7 and R Studio. Correlations were assessed using Spearman’s rank test, and predictors of in-hospital mortality were evaluated with univariate Firth logistic regression and ROC analysis.

Results: The mean age of participants was 64.8 ± 10.5 years; 53.3% were female. The mean NT-proBNP level was 6443 ± 4953 pg/mL, and the mean hospital stay was 10.8 ± 6.4 days. NT-proBNP levels were significantly higher in non-survivors (10977 ± 5687 pg/mL) than survivors (5607 ± 4396 pg/mL; p = 0.027). A moderate positive correlation was observed between NT-proBNP and hospital stay (ρ = 0.425, p = 0.004). ROC analysis identified an NT-proBNP cut-off of 8823 pg/mL for predicting mortality (AUC = 0.76, sensitivity = 86%, specificity = 79%).

Conclusion: Elevated NT-proBNP levels at admission are strongly associated with prolonged hospital stay and increased in-hospital mortality among HF patients. NT-proBNP may serve as a valuable biomarker for early risk stratification and assessing prognosis, enabling efficient resource allocation.

## Introduction

Heart failure (HF) is a progressive debilitating disease that affects patients all over the world, causing symptoms such as shortness of breath, weakness, and congestion due to the heart's inability to supply blood properly, impacting the quality of life, morbidity, and mortality [1]. HF is a prevalent cause of recurrent hospital admissions because of exacerbated symptoms as well as acute decompensation. Therefore, controlling the length and consequences of hospitalization of patients with HF has become an important priority for healthcare professionals, as it has been linked to elevated healthcare expenditure, increased risk of complications, and worse long-term prognosis [2,3].

N-terminal pro-B-type natriuretic peptide (NT-proBNP) is a cardiac biomarker released following myocardial stress, most importantly in HF [4]. It is used extensively in diagnosing HF, quantifying disease severity, and prognosis. High levels of NT-proBNP are associated with the severity of HF symptoms and have been found to predict unfavorable clinical outcomes, such as hospitalization, readmission, and death [5,6]. Although NT-proBNP is well recognized as a valid biomarker for the diagnosis and monitoring of HF progression, its possible utility in predicting certain hospital outcomes, especially duration of stay, is a topic for clinical research. Yet its predictive value as an indicator of hospital outcomes, namely the duration of hospital stay, is not well investigated in the case of HF [7,8].

Understanding the relationship between NT-proBNP levels and the length of hospital stay in patients with HF provides valuable insights for optimizing healthcare resource allocation and guiding treatment approaches. The assessment of NT-proBNP levels among patients who might experience extended hospitalizations enables medical staff to create more targeted interventions leading to better patient outcomes and hospital resource efficiency. People who remain hospitalized with HF have a higher chance of developing hospital-acquired complications and healthcare costs increase, while systems become more burdened. Early detection of patients requiring extended hospital stays potentially enables practitioners to organize timely discharge plans, which reduce hospital admissions and enhance the operational effectiveness of HF care [9,10].

This retrospective study examines how NT-proBNP influences care duration for patients admitted with HF. The research analyzes NT-proBNP test results in combination with hospital stay duration to evaluate the strength of NT-proBNP in predicting health service requirements and treatment results. By investigating NT-proBNP's predictive value for hospital outcomes, this research could provide an evidence-based approach to early intervention and tailored treatment plans, which may translate into improved clinical outcomes and more effective utilization of healthcare resources. This study seeks to promote better risk stratification for improved healthcare outcomes while reducing the tremendous costs involved in HF treatment.

Therefore, this retrospective study aims to evaluate the association between admission NT-proBNP levels and the duration of hospital stay among patients admitted with HF and to identify clinical predictors of elevated NT-proBNP levels and their relationship with in-hospital outcomes, including mortality.

## Materials and methods

This was a hospital-based retrospective observational study, conducted from 20th October 2024 to 20th May 2025. This study was conducted in the Department of General Medicine, Father Muller Medical College Hospital, Mangalore, Karnataka, India.

Study population

All the patients admitted to the hospital for HF, above 18 years of age, and received inpatient care were included in the study. HF was defined according to the European Society of Cardiology (ESC) 2021 criteria [11], based on the presence of typical symptoms (such as dyspnea, orthopnea, or fatigue), clinical signs of volume overload, and objective evidence of cardiac structural or functional abnormality on echocardiography. Patients were further categorized by left ventricular ejection fraction (LVEF) into HF with reduced ejection fraction (HFrEF) (<40%), HF with mid-range ejection fraction (HFmrEF) (40-49%), and HF with preserved ejection fraction (HFpEF) (≥50%), in accordance with current ESC guidelines. However, patients with a history of organ transplant, pregnancy, malignancy, and chronic kidney disease (CKD) on dialysis were excluded.

Sample size

The sample size was determined based on power calculations using the sample size formula for the specific statistical test and assumptions used in the study design. Assuming a two-tailed test for the correlation coefficient, a power of 80%, alpha of 0.05, and a moderate effect size of 0.5, the sample size formula for Pearson's correlation coefficient is as follows:

n = \frac{\left( Z_{\alpha/2} + Z_{\beta} \right)^2}{\ln\ln(1 + r) - \ln(1 - r)^2}

Where n is the required sample size, Zα/2 is the critical value of the standard normal distribution at the desired level of significance (1.96 for alpha = 0.05), Zβ is the critical value of the standard normal distribution at the desired power (0.84 for power = 0.8), and r is the expected correlation coefficient (0.5 for moderate effect size).

Assuming the anticipated correlation coefficient of 0.5, the sample size calculation for a correlation coefficient test was n = 44.70 ≈ 45. Based on this calculation, the sample size required to achieve the desired objective was 45 cases [12].

Ethical considerations

Ethical clearance was obtained from the Institutional Ethics Committee of Father Muller Medical College Hospital, Mangalore. Permission was also granted from the Medical Records Department (MRD) to retrieve relevant patient data. All data were anonymized to maintain confidentiality.

Data collection

Demographic, clinical, and laboratory details were collected from medical records, including NT-proBNP levels, echocardiography findings, comorbid conditions, duration of hospital stay, ICU stay, mortality, and use of ventilatory support. NT-proBNP samples were obtained on admission, prior to initiation of intravenous diuretic therapy, using a chemiluminescent immunoassay calibrated against the manufacturer’s standard reference. HF severity was categorized according to echocardiographic EF groups: HFrEF (<40%), HFmrEF (40-49%), and HFpEF (≥50%).

Comorbidities recorded included hypertension, diabetes mellitus, CKD not on dialysis, chronic liver disease, chronic obstructive pulmonary disease, and documented coronary artery disease based on prior angiography or echocardiographic wall-motion abnormalities. During hospitalization, all patients received medications according to hospital protocol, including diuretics, ACE inhibitors or ARNI, beta-blockers, aldosterone antagonists, and vasodilators, individualized according to patient needs, unless contraindicated. However, detailed medication data and data on comorbidity were not consistently available for retrospective analysis.

Statistical analysis

The collected data were coded and entered into a Microsoft Excel (Microsoft Corp., Redmond, WA) datasheet, and all analyses were performed using Jamovi Version 2.7 and R Studio (Posit, Boston, MA). Categorical variables were summarized as frequencies and percentages, while continuous variables were assessed for normality using the Shapiro-Wilk test. Normally distributed variables were expressed as mean ± standard deviation (SD) and compared using the independent sample t-test, whereas non-normally distributed data were expressed as median (interquartile range) and compared using the Mann-Whitney U test. Associations between categorical variables were analysed using the chi-square test or Fisher’s exact test where appropriate.

The relationship between NT-proBNP levels and length of hospital stay was evaluated using Spearman’s rank correlation. Univariate Firth logistic regression was applied to identify predictors of in-hospital mortality, and receiver operating characteristic (ROC) curve analysis was performed to determine the discriminatory power and optimal cut-off values of significant continuous predictors, expressed as area under the curve (AUC) with corresponding sensitivity and specificity. Effect sizes were reported as Cohen’s d or correlation coefficient (r) where relevant. Graphical representations, including forest plots and correlation heatmaps, were generated in R Studio. A two-tailed p-value ≤0.05 was considered statistically significant.

Because only seven in-hospital deaths occurred, multivariable logistic regression was avoided to prevent model overfitting. Instead, univariate Firth logistic regression was employed to obtain bias-reduced estimates suitable for small-sample, rare-event data. Adjustment for multiple comparisons was not performed, as this exploratory study was designed to generate hypotheses for validation in future larger cohorts.

## Results

**Figure 1:**
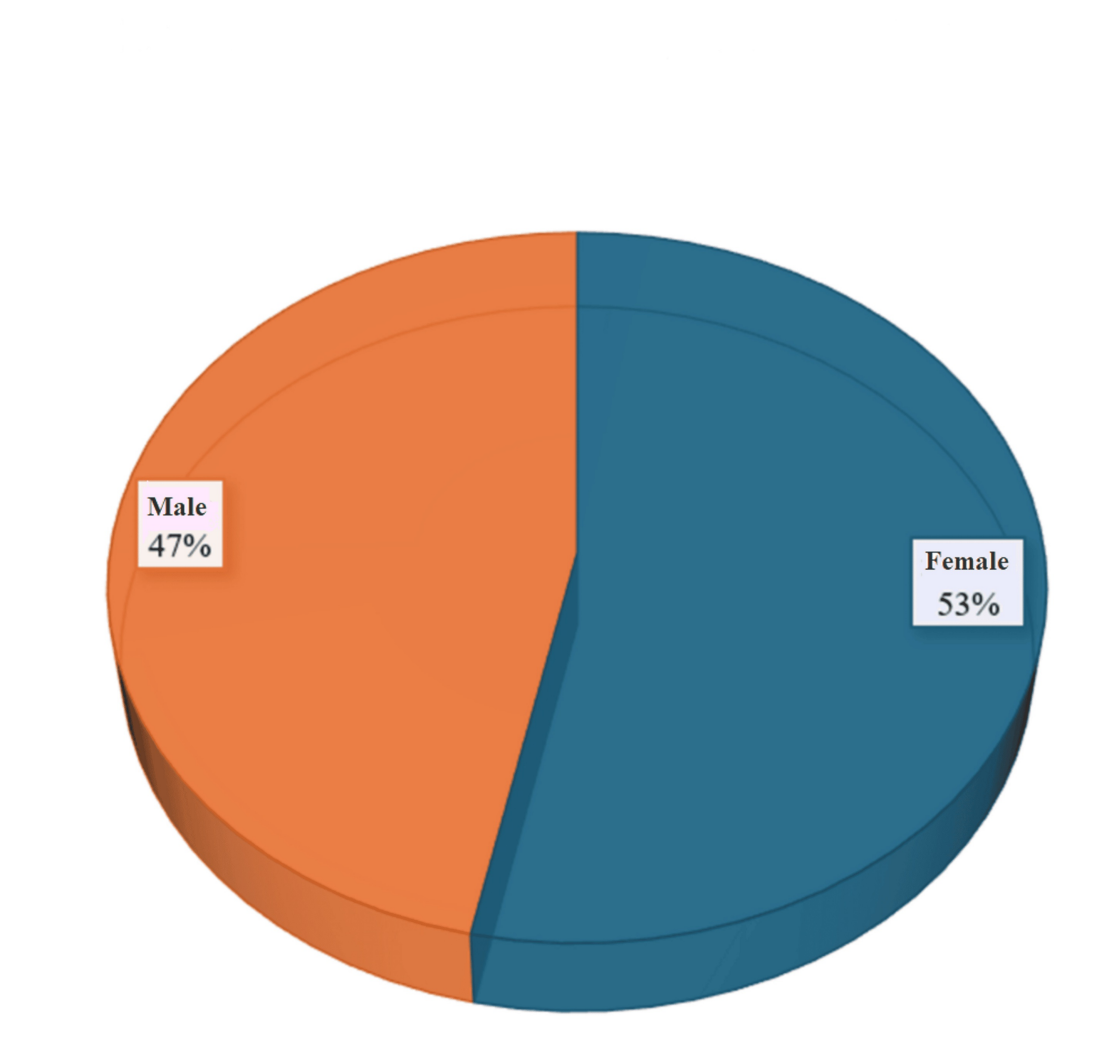
Distribution of study population by gender.

**Figure 2:**
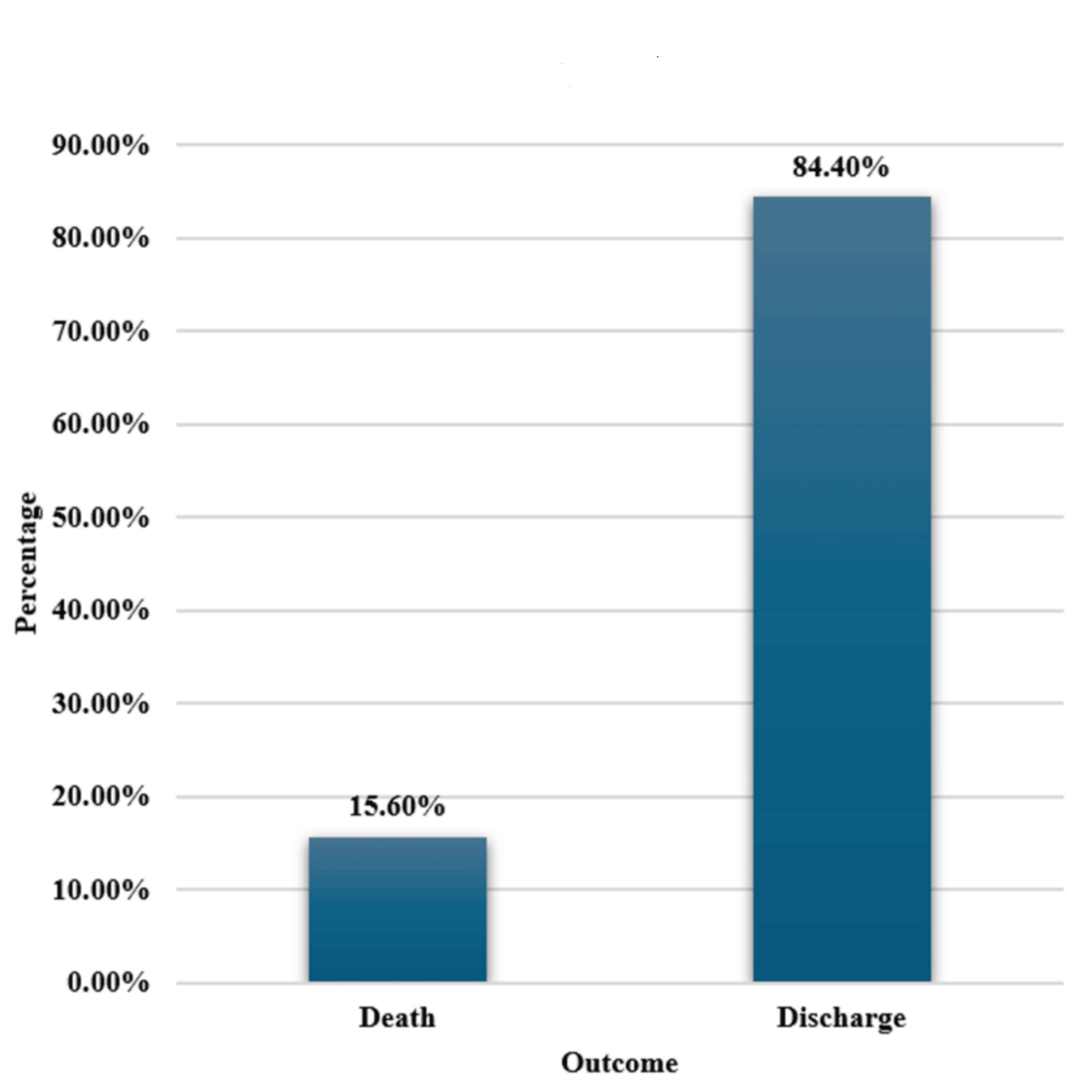
Distribution of study population by outcome of disease.

Among 45 participants, the average age was 64.8 ± 10.5 years (range: 35-85), and 53.3% of the participants were female (Figure 1). The most common comorbidities were diabetes (71.1%) and hypertension (75.6%), with just one patient having chronic liver disease (CLD) and chronic renal disease (24.4%) (Table 1). In total, 38 patients (84.4%) were discharged from the hospital, while seven patients (15.6%) died (Figure 2).

**Table 1:** Frequency distribution of study variables. CLD: chronic liver disease; COPD: chronic obstructive pulmonary disease; CKD: chronic kidney disease; MI: myocardial infarction.

Variable	Category	Number	Percentage
Sex	Female	24	53.3
	Male	21	46.7
Outcome	Death	7	15.6
	Discharge	38	84.4
Cardiac chambers	Abnormal	36	80.0
	Normal	9	20.0
Cardiac wall motion abnormality	Abnormal	21	46.7
	Normal	24	53.3
Hypertension	Yes	34	75.6
	No	11	24.4
Diabetes	Yes	32	71.1
	No	13	28.9
Smoking	Yes	20	44.4
	No	25	55.6
Alcohol	Yes	15	33.3
	No	30	66.7
CKD (not on dialysis)	Yes	11	24.4
	No	34	75.6
CLD	Yes	1	2.2
	No	44	97.8
Cause of death	COPD	1	2.2
	MI	6	13.4
	Nil	38	84.4

The distribution of continuous variables is displayed in Table 2. With a range of 35-85, the average age was 64.8 years. With a mean NT-proBNP of 6443 pg/mL, patients varied greatly (528-19800). The mean ICU stay was 5.7 days, and an average hospital stay was 10.8 days. The mean LVEF was 36%. The mean hemoglobin was 10.8 g/dL, and the mean creatinine was 1.4 mg/dL. The average duration of non-invasive ventilation (NIV) was 4.2 days, whereas the average duration of invasive ventilation was 1.5 days.

**Table 2:** Descriptive statistics of continuous variables. NT-proBNP: N-terminal pro-B-type natriuretic peptide; EF: ejection fraction; NIV: non-invasive ventilation; ICU: intensive care unit.

Variable	Mean ± SD	Median (IQR)	95% CI of mean	Min–max
Age (years)	64.8 ± 10.5	64 (15)	61.7–68.0	35–85
NT-proBNP (pg/mL)	6443 ± 4953	5680 (8144)	4955–7931	528–19800
Hospital stay (days)	10.8 ± 6.4	10 (8)	8.9–12.7	2–29
EF (%)	36.0 ± 10.3	36 (12)	32.9–39.1	16–58
ICU stay (days)	5.7 ± 4.5	5 (8)	4.3–7.1	0–20
Hemoglobin (g/dL)	10.8 ± 1.6	10.8 (1.9)	10.3–11.2	5.6–14.2
Creatinine (mg/dL)	1.4 ± 1.3	1.0 (0.6)	1.0–1.8	0.4–7.7
NIV (days)	4.2 ± 3.9	4 (5)	3.1–5.4	0–18
Ventilator (days)	1.5 ± 2.6	0 (2)	0.7–2.3	0–10

Based on EF criteria, 27 patients (60.0%) had HFrEF (<40%), 13 (28.9%) had HFmrEF (40-49%), and 5 (11.1%) had HFpEF (≥50%). The mean NT-proBNP levels were highest among patients with HFrEF (7493 ± 4858 pg/mL), followed by HFpEF (5036 ± 5228 pg/mL) and HFmrEF (4800 ± 4844 pg/mL). This pattern indicates progressively rising NT-proBNP with worsening systolic dysfunction, which is displayed in Table 3.

**Table 3:** Distribution of NT-proBNP across HF subtypes based on EF. HF: heart failure; EF: ejection fraction; NT-proBNP: N-terminal pro-B-type natriuretic peptide.

HF subtype	n (%)	Mean NT-proBNP (pg/mL) ± SD
HFrEF (<40%)	27 (60.0%)	7493.9 ± 4858.3
HFmrEF (40–49%)	13 (28.9%)	4800.0 ± 4844.2
HFpEF (≥50%)	5 (11.1%)	5036.2 ± 5228.2

**Figure 3:**
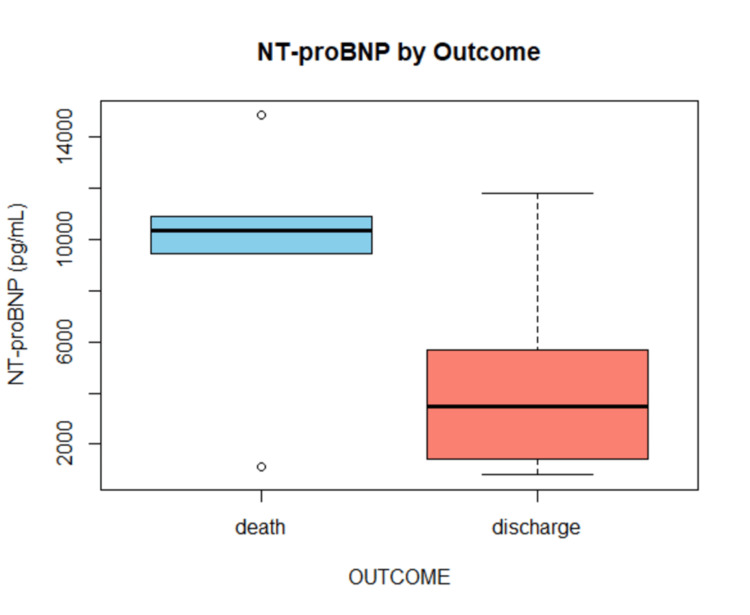
NT-proBNP levels by outcome. NT-proBNP: N-terminal pro-B-type natriuretic peptide.

NT-proBNP levels were substantially higher in deceased patients (mean 10,977 ± 5687 pg/mL) than in discharged patients (mean 5607 ± 4396 pg/mL) (Figure 3). NT-proBNP levels peaked in the 61-70 age range (mean 8730 pg/mL), and they tended to increase with age. NT-proBNP was higher in those with a lower EF (<40%) (7494 ± 4858 pg/mL) than in those with an EF of ≥40% (4866 ± 4797 pg/mL). Similarly, NT-proBNP levels were nearly twice as high in patients with longer ICU hospitalizations (>5 days) (9577 ± 4602 pg/mL) compared to those with shorter stays (≤5 days: 3935 ± 3671 pg/mL), as shown in Table 4.

**Table 4:** Comparison of NT-proBNP by outcome, age, EF, and ICU stay. NT-proBNP: N-terminal pro-B-type natriuretic peptide; EF: ejection fraction; ICU: intensive care unit.

Group	Subgroup	NT-proBNP (max–min)	Mean ± SD
Outcome	Death	19800–1100	10977.1 ± 5686.7
	Discharge	15800–528	5607.2 ± 4395.7
Age	<50 years	3630–890	2626.7 ± 1510.0
	51–60 years	14200–528	6138.4 ± 4449.7
	61–70 years	19800–839	8730.4 ± 5946.2
	71–80 years	13000–797	5224.9 ± 4448.4
	>81 years	7320–5680	6500.0 ± 1159.7
EF	<40%	19800–663	7493.9 ± 4858.3
	≥40%	15800–528	4865.6 ± 4796.7
ICU stay	0–5 days	15800–528	3935.2 ± 3671.2
	>5 days	19800–663	9576.7 ± 4601.7

Table 5 provides a summary of the clinical determinants of in-hospital death. Serum creatinine (1.6 vs. 1.0 mg/dL, p = 0.034), ejection fraction (28% vs. 36%, p = 0.013), and NT-proBNP levels (12,000 vs. 5757 pg/mL, p = 0.027) were also considerably higher in non-survivors. The greatest predictor was ventilator support: deaths necessitated greater NIV support (6 vs. 4 days, p = 0.049) and longer ventilator usage (6 vs. 0 days, p < 0.001). One non-survivor had CLD, although it was not statistically significant.

**Table 5:** Significant clinical predictors of in-hospital mortality. NT-proBNP: N-terminal pro-B-type natriuretic peptide; EF: ejection fraction; CLD: chronic liver disease. Bold value indicates statistically significant results.

Variable	Overall mean ± SD	Median (IQR)	Test	Effect size	p-value
NT-proBNP (pg/mL)	6443 ± 4953	5680 (8144)	Mann–Whitney U	r = –0.33	0.027
EF (%)	36 ± 10.3	36 (12)	Welch’s t-test	d = –0.92	0.013
Creatinine (mg/dL)	1.4 ± 1.3	1.0 (0.6)	Welch’s t test	d = –0.55	0.034
NIV (days)	4.2 ± 3.9	4 (5)	Mann–Whitney U	r = –0.29	0.049
Ventilator (days)	1.5 ± 2.6	0 (2)	Mann–Whitney U	r = –0.49	0.001
CLD	–	–	χ² = 0.92 ; OR = 76	–	0.336

**Figure 4:**
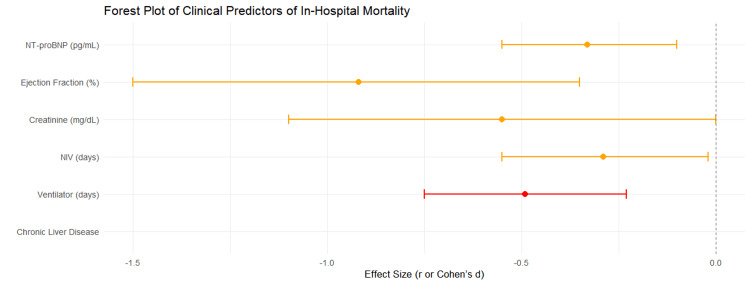
Forest plot of clinical predictors of in-hospital mortality. NT-proBNP: N-terminal pro-B-type natriuretic peptide.

These findings are displayed as a Forest plot of effect sizes with 95% CIs in Figure 4. Reduced EF (Cohen's d = -0.92, p = 0.013) and NT-proBNP (r = -0.33, p = 0.027) were less affected than ventilator time (r = -0.49, p < 0.001). CLD had no discernible effect, whereas creatinine and NIV days did. All these results show that elevated NT-proBNP, decreased cardiac function, renal dysfunction, and increased ventilatory needs are significant predictors of poor in-hospital outcomes.

**Figure 5:**
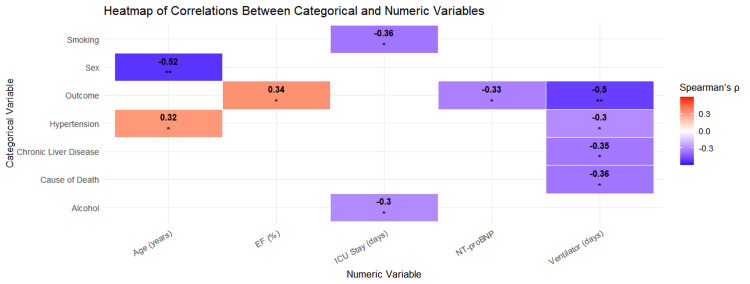
Correlation heatmap of categorical and continuous clinical parameters. EF: ejection fraction.

Significant correlations between categorical and numerical clinical indicators are depicted in the correlation heatmap (Figure 5). Spearman's correlation coefficients (ρ) are shown by colors, with blue denoting a negative correlation and red denoting a positive correlation. While smoking and alcohol consumption were associated with longer intensive care unit (ICU) hospitalizations and CLD with increased ventilator usage, outcome was correlated with NT-proBNP, EF, and ventilator days. Weak (<0.3), moderate (0.3-0.5), and strong (>0.5) correlations were interpreted.

The findings of univariate Firth logistic regression for in-hospital mortality factors are displayed in Table 6. Higher probabilities of mortality were linked to each incremental increase in NT-proBNP, which was a meaningful predictor (OR 1.0002, 95% CI 1.0000-1.0004, p = 0.012). Additionally, mortality was predicted by reduced EF (OR 0.91, 95% CI 0.81-0.99, p = 0.031), meaning that every 1% drop in EF raised the chance of dying. The strongest correlation was found for ventilator days (OR 1.55, 95% CI 1.18-2.22, p = 0.001). Creatinine did not exhibit a statistically significant effect, although NIV duration and CLD did indicate borderline significance.

**Table 6:** Univariate Firth logistic regression for predictors of in-hospital mortality. NT-proBNP: N-terminal pro-B-type natriuretic peptide; EF: ejection fraction; CLD: chronic liver disease; NIV: non-invasive ventilation. Bold value indicates statistically significant results.

Predictor	Estimate (SE)	Odds ratio (95% CI)	p-value
NT-proBNP (pg/mL)	0.0002 (0.00009)	1.0002 (1.0000–1.0004)	0.012
EF (%)	–0.098 (0.049)	0.91 (0.81–0.99)	0.031
Creatinine (mg/dL)	–0.222 (0.391)	0.80 (0.04–1.40)	0.556
NIV (days)	–0.291 (0.173)	0.75 (0.48–1.02)	0.067
Ventilator (days)	0.437 (0.150)	1.55 (1.18–2.22)	0.001
CLD	2.877 (1.687)	17.8 (0.85–2714)	0.063

**Figure 6:**
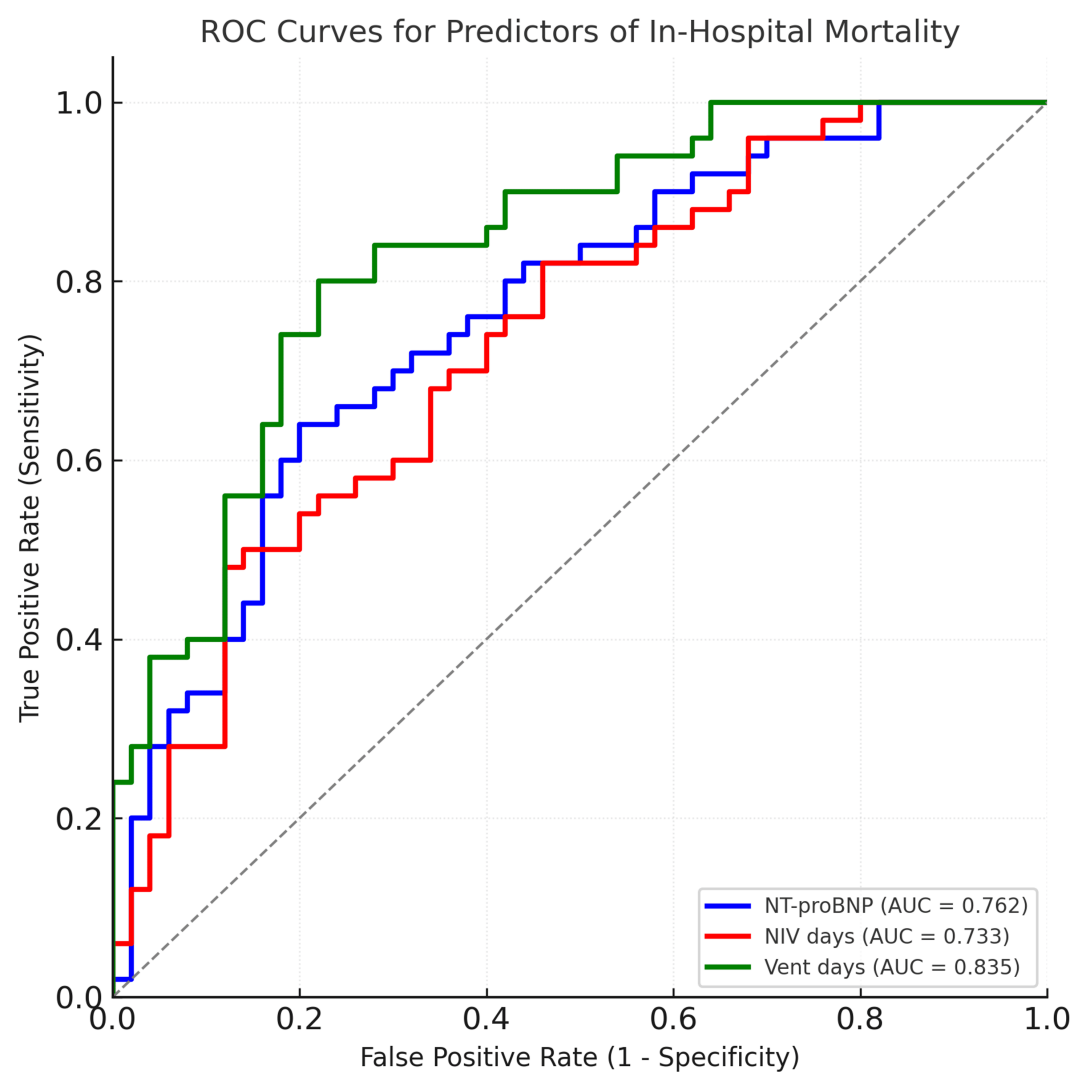
ROC analysis of significant continuous predictors for in-hospital mortality. ROC: receiver operating characteristic.

The ROC analysis of significant continuous predictors for in-hospital mortality is summarized in Table 7 and Figure 6. At an ideal cut-off of 8823 pg/mL, NT-proBNP showed good discrimination (AUC 0.76, 95% CI 0.54-0.99, p = 0.01), with 86% sensitivity and 79% specificity. NIV length demonstrated low specificity (47%) and excellent sensitivity (100%) with little discrimination (AUC 0.73, p = 0.002). With high sensitivity (86%) and specificity (74%), ventilator days had the highest predictive value (AUC 0.84, 95% CI 0.66-1.00, p < 0.001).

**Table 7:** ROC analysis of significant continuous predictors for in-hospital mortality. AUC: area under the curve; PPV: positive predictive value; NPV: negative predictive value; NT-proBNP: N-terminal pro-B-type natriuretic peptide; NIV: non-invasive ventilation; ROC: receiver operating characteristic.

Predictor	AUC	95% CI	Z value	P value	Cut-off	Sensitivity	Specificity	Accuracy	PPV	NPV
NT-proBNP (pg/mL)	0.763	0.54–0.99	2.31	0.01	8823	0.857	0.789	0.8	0.429	0.968
NIV (days)	0.733	0.57–0.90	2.81	0.002	4.5	1.000	0.474	0.64	0.263	0.923
Ventilator (days)	0.838	0.66–1.00	3.71	0.001	0.5	0.857	0.737	0.76	0.375	0.966

**Figure 7:**
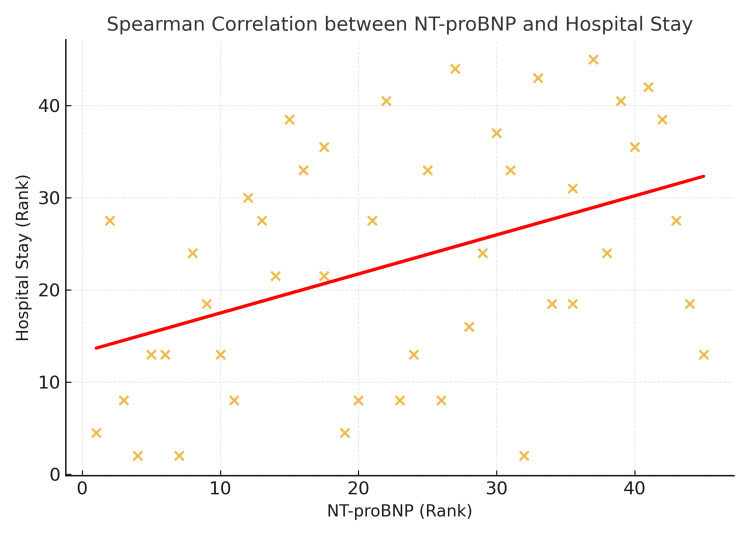
Spearman correlation between NT-proBNP and hospital stay. NT-proBNP: N-terminal pro-B-type natriuretic peptide.

The scatter plot (Figure 7) shows ranked NT-proBNP values against ranked hospital stay durations, with a red line representing the fitted linear trend. A moderate, statistically significant positive correlation was observed (ρ = 0.425, p = 0.004), indicating that higher NT-proBNP levels were associated with longer hospital stays.

## Discussion

One of the biggest challenges of modern medicine is detecting silent cardiac damage to stratify cardiac risk. Several studies have established that NT-proBNP is an important biomarker mainly synthesized and secreted by myocytes in the left ventricle (LV) as a response to myocytes stretched by pressure overload or volume expansion of the ventricle and is a powerful predictor of cardiovascular outcomes. In this retrospective study, our aim was to evaluate the association between admission NT-proBNP levels and the duration of hospital stay in patients with HF and to identify clinical predictors of elevated NT-proBNP and adverse in-hospital outcomes. Our findings demonstrated that higher NT-proBNP levels were significantly associated with longer hospitalization and increased mortality, consistent with previous reports highlighting its role as a powerful prognostic marker in HF [13].

In this study, it was found that the level of NT-proBNP in deceased patients was roughly twice as high as that of survivors (mean ~10,977 vs ~5,607 pg/mL) and that higher NT-proBNP correlated with older age and reduced LVEF. Additionally, it was also found that every additional point rise in NT-proBNP modestly increased odds of death (OR 1.0002 per pg/mL), reflecting a strong graded relationship between NT-proBNP and mortality risk. These findings are consistent with prior studies. Welsh et al. in their study established a significant association between increasing age and elevated NT-proBNP levels [14]. Nakano et al. concluded that in patients with normal creatinine, NT-proBNP levels correlated significantly with EF. Furthermore, it was also noted that mortality was high in the group with the highest NT-proBNP [15].

In our cohort, NT-proBNP concentrations demonstrated a stepwise increase across HF subtypes, with the highest levels observed in HFrEF patients and progressively lower values in HFmrEF and HFpEF, reinforcing the inverse relationship between NT-proBNP and systolic function and consistency with prior studies demonstrating higher biomarker levels in patients with reduced EF [16].

Higher NT-proBNP was associated with longer and more complicated hospital courses. Patients who stayed in the ICU for more than five days had nearly twice the NT-proBNP of those with shorter stays (mean ~9577 vs. ~3935 pg/mL), and logistic regression identified NT-proBNP and duration of mechanical ventilation as the strongest mortality predictors. In practical terms, an elevated NT-proBNP at admission signals a high likelihood of prolonged ICU care and need for respiratory support. In our study, ventilator usage was itself a powerful marker of mortality. Consistently, the ROC curve for ventilator days yielded the highest AUC (0.84) for predicting death. NT-proBNP's good discrimination (AUC≈0.76) and an optimal cut-off of ~8823 pg/mL in our analysis suggest it can be used to stratify risk: levels above this threshold should alert clinicians to very high short-term mortality risk. Patients who required invasive ventilation had much higher in-hospital death rates, echoing Jentzer et al.'s observation that HF patients admitted with shock or requiring ventilation/vasopressors are "at particularly high risk of death" [17]. Similarly Udani et al. also found that in quartile with a high level of NT-proBNP, there was increased lengths of hospital stay [12].

In this study, Spearman’s rank correlation analysis was performed to evaluate the relationship between NT-proBNP levels and length of hospital stay. There was a moderate positive correlation between the two variables (ρ = 0.425, p = 0.004), indicating that higher NT-proBNP levels were associated with longer durations of hospitalization. This suggests that patients with more elevated NT-proBNP values at presentation tend to have more prolonged admissions, likely reflecting greater illness severity. Savarese et al. reported that reductions in natriuretic peptide concentrations were associated with a 32% lower risk of HF-related hospitalizations across 19 trials. This supports our observation that elevated NT-proBNP levels correlate with prolonged hospitalization, reflecting more severe disease and higher resource utilization [18]. Additionally, López-Vilella et al. concluded that variables such as creatinine and NT-ProBNP at hospital admission may define a subgroup of patients who will likely have a longer hospital stay [19].

While our study was limited by its smaller sample size and single-center design, our findings align with evidence from larger multicenter cohorts. Notably, Udani et al. conducted a retrospective analysis of 21,445 patients with HF and demonstrated that the quartile with higher admission NT-proBNP levels was significantly associated with longer hospital stays, increased in-hospital mortality, and higher 60- and 90-day readmission rates. Their results showed a clear dose-response pattern across NT-proBNP quartiles, highlighting its strong prognostic value. Although our study is less extensive, the consistency in observed trends reinforces the clinical relevance of admission NT-proBNP as a marker of disease severity and adverse outcomes [12].

Clinically, these results reinforce that an elevated NT-proBNP in HF should trigger aggressive management and careful monitoring. We extend this by showing that very high NT-proBNP (on the order of 8-12×10^3^ pg/mL) on admission may result in poor in-hospital outcome as well as prolonged hospital stay. In practice, a patient with such elevated NT-proBNP and reduced EF likely has severe systolic dysfunction and congestion. This should prompt optimization of guideline-directed therapy (diuretics, vasodilators, inotropes, or device therapy as indicated) and possibly early critical care involvement. Consistently, Nazemiyeh et al. found obstructive and restrictive ventilatory impairments in patients with HF as well as a significant correlation between the severity of HF and pulmonary dysfunction [20].

In summary, NT-proBNP can inform prognosis: very high values could be used as a criterion for ICU admission or closer surveillance, as well as it might indicate prolonged hospital stay and problems associated with it, while lower values might reassure that aggressive escalation (e.g. mechanical ventilation) may not be immediately needed. Likewise, reduced EF (<40%) in our cohort independently raised mortality odds, highlighting that concomitant severe systolic dysfunction should amplify clinical concern in the face of elevated NT-proBNP. These separate indications may have a synergistic effect on mortality and hospital stay.

Our study has important limitations. As a retrospective single-center analysis, it is subject to selection bias and unmeasured confounding. Because of the small number of in-hospital deaths, multivariable analysis was not feasible, which limits the ability to adjust for potential confounding factors. The sample size is relatively small (n = 45) with few deaths (n = 7), so estimates are imprecise, and the findings may not generalize to other settings or patient populations. Key clinical variables, such as details of comorbidities, guideline-directed medical therapy, and treatment protocols followed during hospitalization, were not uniformly available from patient records and could not be comprehensively analyzed. Information regarding coronary artery disease status or revascularization therapy was incomplete in medical records and therefore could not be analyzed in relation to mortality outcomes. Additionally, the wide standard deviations observed in NT-proBNP and hospital stay reflect the biological and clinical heterogeneity of HF patients, which is common in real-world retrospective cohorts and may influence the precision of mean estimates. Only admission NT-proBNP was analyzed, so dynamic changes during hospitalization were not captured. The classification of HF severity was based solely on EF without NYHA functional class data, which could have provided additional context regarding clinical status. Finally, the ROC-derived cut-off (8823 pg/mL) is specific to our cohort and requires external validation.

## Conclusions

This retrospective single-center study demonstrates that admission NT-proBNP levels are strongly associated with adverse in-hospital outcomes among patients with HF. Elevated NT-proBNP levels correlated with increased mortality, prolonged ICU stay, extended duration of mechanical ventilation, and longer overall hospitalizations. Patients presenting with very high NT-proBNP levels (approximately 8000-12000 pg/mL) had significantly poorer prognosis, and an optimal cut-off of 8823 pg/mL showed good discriminatory ability for predicting mortality. These findings align with existing evidence from larger multicenter cohorts and support the role of NT-proBNP as a reliable biomarker for early risk stratification and prognostication in HF.

Since this study is retrospective single-center study with a small sample size, further prospective studies involving large multicenter cohorts are warranted to validate the findings. It is also important to evaluate dynamic changes in NT-proBNP during hospitalization to enhance prognostic accuracy, and further research is required in this direction to establish the correlation. Early identification of high-risk patients through NT-proBNP measurement could facilitate timely escalation of care, optimize resource allocation, and ultimately improve clinical outcomes in patients admitted with HF.
